# Efficient Azimuth Image-Quality Prediction for Block-Design in Squinted Spotlight SAR Imaging

**DOI:** 10.3390/s26196252

**Published:** 2026-10-02

**Authors:** Boyeon Koh, Dongjun Lee, Ungdai Ko

**Affiliations:** Agency for Defense Development (ADD), Daejeon 34060, Republic of Korea; junman882@naver.com (D.L.); higrandeur@gmail.com (U.K.)

**Keywords:** synthetic aperture radar (SAR), squint, image-quality prediction, azimuth resolution, PSLR, ISLR, block-processing planning

## Abstract

This paper presents an efficient image-quality prediction method for planning block processing in squinted spotlight synthetic aperture radar (SAR) imaging. Before real SAR data are divided into processing blocks, candidate processing-reference locations and feasible block extents must be evaluated. Repeated evaluation using a full two-dimensional focusing processor is computationally expensive and is therefore inefficient for iterative planning. To overcome this problem, first, a new one-dimensional azimuth-compression kernel is derived by applying the principle of stationary phase (POSP) to the azimuth-phase component of the SAR signal. Second, the possibility of replacing the full two-dimensional ω–k kernel with the derived kernel is mathematically analyzed by examining the residual quadratic phase and its range-frequency dependence. Third, applying the derived kernel to initial block design substantially reduces the computation time. In the present study, idealized platform trajectory and ground-surface conditions are assumed. Under these assumptions, the proposed kernel predicts azimuth resolution, peak sidelobe ratio (PSLR), and integrated sidelobe ratio (ISLR). These metrics are then used as image-quality criteria for initial block design. Simulations using 36 point targets distributed over a 1.5 km × 1.5 km test area demonstrate that the proposed 1D kernel closely matches the image quality obtained using the full ω–k kernel. These results demonstrate that the proposed 1D azimuth-compression kernel can efficiently replace full ω–k processing during initial block design.

## 1. Introduction

High-resolution squinted synthetic aperture radar (SAR) imaging over a wide scene is challenging because range migration, range–azimuth coupling, and spatially varying phase errors increase with scene extent. Previous studies have mainly focused on improving the focusing processor itself, including extended two-step and frequency-scaling methods [1,2], extended or nonlinear chirp-scaling methods [3,4,5], and modified Stolt mapping, effective-wavelength processing, and time-domain rotation [6,7,8]. Another line of research has considered spatial partitioning or block processing, in which block dimensions are determined using phase-error limits, iterative partitioning, or space-variant post-filtering [9,10,11,12].

At the initial block-design stage, the number of processing blocks, processing-reference locations, feasible block extents, and expected image quality are not known in advance. Before performing final block design using fully focused SAR data, these design variables need to be explored to obtain reasonable initial estimates. If this preliminary exploration relies on repeatedly applying a computationally expensive full focusing algorithm, such as the two-dimensional ω–k processor, the design process can become inefficient. This two-stage design strategy is often adopted because parameters obtained under idealized conditions cannot be directly used for final block design with real SAR data. The initial design therefore provides practical starting values and feasible design ranges, which can subsequently be refined using actual SAR data while accounting for scene-dependent effects.

The first contribution of this work is a newly derived one-dimensional azimuth-compression kernel. The kernel is obtained by applying the principle of stationary phase (POSP) to the azimuth-phase component of the SAR signal and is intended for use during the initial block-design stage.

The second contribution is a mathematical analysis of the possibility of replacing the full two-dimensional ω–k kernel with the derived one-dimensional kernel. This analysis examines the residual quadratic phase and its range-frequency dependence to assess the validity of the proposed replacement.

The third contribution is the application of the derived kernel to initial block design, which substantially reduces the computation time required for iterative evaluation.

Finally, simulations using 36 distributed point targets over a 1.5 km × 1.5 km test area demonstrate that the proposed one-dimensional kernel closely matches the image quality obtained using the full ω–k kernel. These results demonstrate that the proposed kernel can efficiently replace full ω–k processing during initial block design.

## 2. Mathematical Model

In practical block design using real SAR data, an initial block layout is generally required before the final block-processing configuration can be determined. This initial layout is typically estimated over the entire imaging area using the acquisition geometry and other known system information. A major difficulty at this stage is that there is no obvious criterion for determining how the scene should be divided into processing blocks. For this reason, an idealized point-target simulation is often performed by assuming a nominal platform trajectory and simplified ground conditions. A processing reference is first selected, and the full ω–k kernel is applied over the imaging area. Additional processing references are then introduced in regions where acceptable focusing is not achieved, and the focusing process is repeated. This procedure continues until the imaging area is sufficiently covered by regions satisfying the required image-quality criteria. The resulting block layout provides practical guidance for subsequent block processing of real SAR data. However, because the full two-dimensional processing kernel must be repeatedly executed for multiple candidate references, this initial design procedure can require substantial computational time. To reduce this burden, the present study introduces a one-dimensional processing kernel for rapid initial block-design analysis and theoretically examines the applicability of the proposed dimensional reduction.

### 2.1. Ground-Coordinate Grid and Imaging Geometry

**Figure 1:**
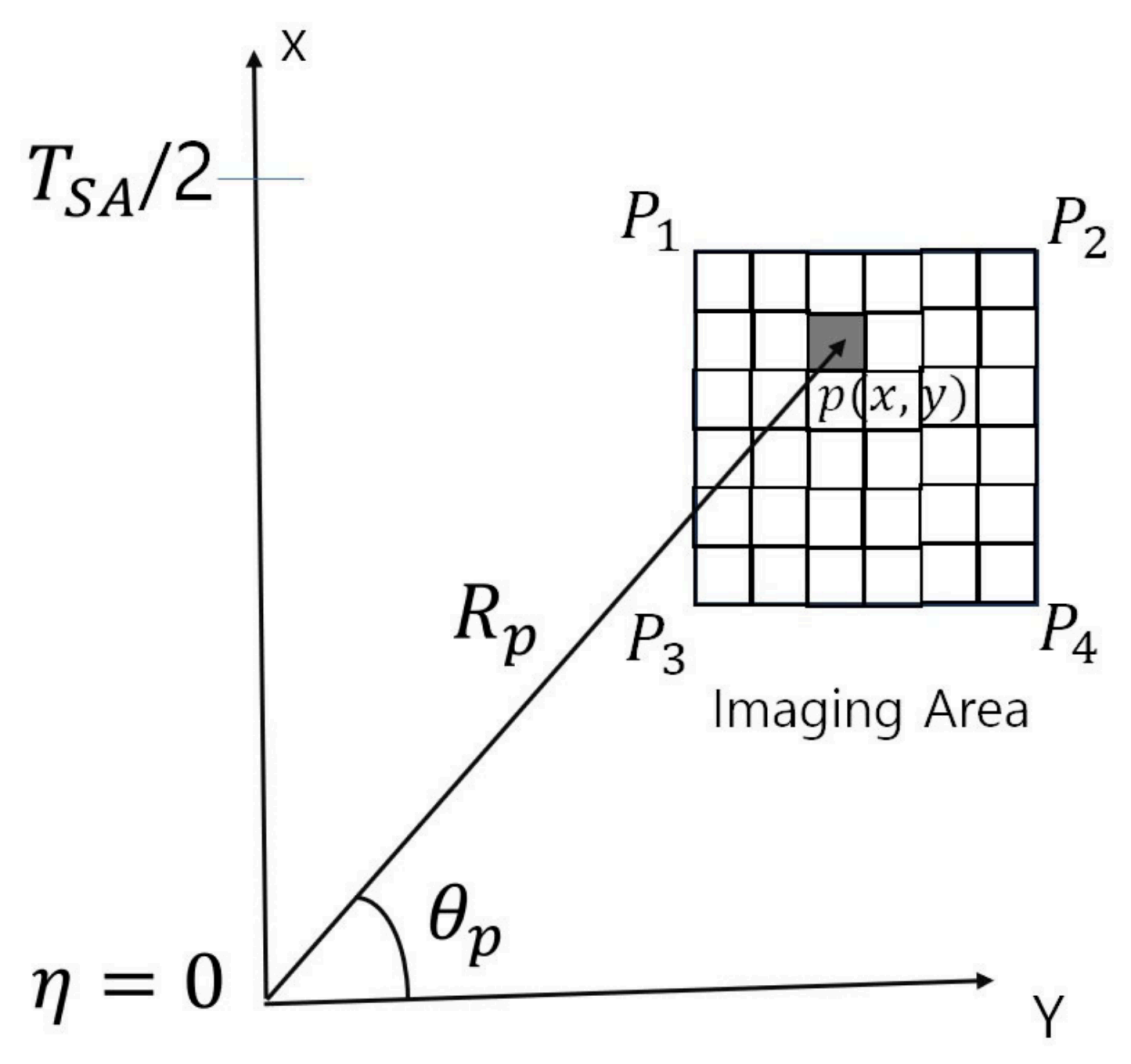
Squinted spotlight SAR imaging geometry with uniformly divided small ground cells. The grey cell represents an arbitrary target point located at *p*(*x*,*y*) within the imaging area, and the arrow indicates the slant range vector from the radar position at *η* = 0 to this point, defined by the slant range *R_p_* and the squint angle *θ_p_*.

The proposed method uses a uniform ground-coordinate grid, while the focusing geometry of each cell is described by its slant range and squint angle. As illustrated in Figure 1, the platform moves along the x-axis over the centered synthetic-aperture interval, *η* ∈ [−*T_SA_*/2, *T_SA_*/2], and its position is expressed as

(1) $$r_{s}\left(\eta \right)={\left[x_{s}\left(\eta \right),\;\;y_{s},\;\;h\right]}^{T},x_{s}\left(\eta \right)=x_{s}\left(0\right)+\mathrm{V\eta}$$

where *V* and *h* denote the platform velocity and altitude. For a point target located at the ground position *p* = (*x*,*y*,0), the corresponding slant range and squint angle are

(2) $$R\left(x,y,\eta \right)=\sqrt{\left[x-x_{s}(\eta ){]}^{2}+(y-y_{s}{)}^{2}+h^{2}\right.}$$

(3) $$\theta (x,y,\eta )=\tan^{-1}\left[\frac{x-x_{s}\left(\eta \right)}{\sqrt{{\left(y-y_{s}\right)}^{2}+h^{2}}}\right]$$

The slant range and squint angle of point target p at the aperture center are defined as *R_p_* = *R*(*x*,*y*,0) and *θ_p_* = *θ*(*x*,*y*,0).

The four corners of the ground imaging area are denoted by *P_i_* = (*x_i_*,*y_i_*), *i* = 1, …, 4. The squint angle is determined by the along-track component of the line-of-sight vector. The ground imaging area is uniformly divided into small rectangular cells, and the center of each cell serves both as an evaluation point and as a candidate block-reference point.

### 2.2. Full ω–k Chain with Squint Stolt Mapping

Based on the effective-wavelength concept for representing the range-frequency dependence of the squint geometry, as introduced in [7], the two-dimensional frequency-domain spectrum of a point target is reformulated to make it suitable for the present study. This reformulation is obtained by applying the principle of stationary phase to the azimuth phase function. Ignoring the range and azimuth weighting terms, the point-target spectrum is given by

(4) $$S_{p}\left(f_{\tau },f_{a}\right)\;∼\exp\left\{-j\frac{4\pi R_{p}}{c}G_{p}\left(F,f_{a}\right)\right\}\exp\left\{-j\frac{\pi f_{\tau }^{2}}{K_{r}}\right\}$$

where *f_τ_* and *f_a_* denote the range and azimuth frequencies, *R_p_* is the slant range of target *p*, *K_r_* is the range chirp rate, and *c* is the speed of light. The function *G_p_*(*F*,*f_a_*) represents the squint-dependent two-dimensional spectral geometry of the target and is defined as follows:

(5) $$G_{p}\left({F,f}_{a}\right)=F\left[q\left(F,f_{a}\right)\sin\theta_{p}+\cos\theta_{p}\sqrt{1-q^{2}\left(F,f_{a}\right)}\right]$$

(6) $$q\left(F,f_{a}\right)=\sin\theta_{0}+\frac{cf_{a}}{2\mathrm{FV}}$$

where *F* = *f_c_* + *f_τ_* denotes the total range frequency, *θ_p_* is the squint angle of target *p*, and *q*(*F*,*f_a_*) denotes the common normalized Doppler variable. Here, *f_a_* is the common baseband azimuth frequency obtained after removing the scene-center Doppler centroid (2*FV*/*c*)*sinθ*_0_. The forms presented in Equations (4) and (5) were specifically derived for the present one-dimensional azimuth-focusing framework by identifying the azimuth-dependent phase term and applying the principle of stationary phase (POSP).

All processing blocks share the azimuth-frequency axis defined with respect to the scene-center Doppler reference. Therefore, *q*(*F*,*f_a_*) uses *sinθ*_0_, whereas the block-specific geometry is incorporated through *θ_b_* in *G_b_*(*F*,*f_a_*). For the proposed one-dimensional azimuth-focusing kernel, the normalized Doppler variable in (6) is evaluated at the center range frequency, *f_τ_* = 0, or equivalently *F* = *f_c_*, as

(7) $$q^{1D}\left(f_{a}\right)=\sin\theta_{0}+\frac{cf_{a}}{2f_{c}V}$$

Consequently, *q*^1*D*^(*f_a_*) depends only on the azimuth frequency and provides the one-dimensional counterpart of the two-dimensional variable *q*(*F*,*f_a_*) in (6).

**Figure 2:**
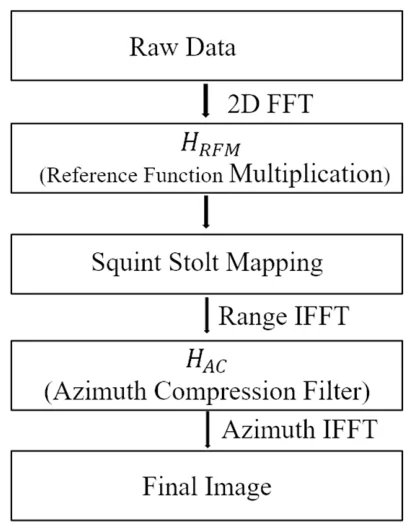
Full ω–k processing chain based on SSM.

Figure 2 shows the full *ω* − *k* chain with squint Stolt mapping. It begins with a two-dimensional Fourier transform, followed by application of the reference-function multiplication filter

(8) $$H_{\mathrm{RFM},b}\left(F,f_{a}\right)=\exp\left\{j\frac{4\pi R_{b}}{c}\left[G_{b}\left(F,f_{a}\right)-F\right]\right\}\exp\left(j\frac{\pi f_{\tau }^{2}}{K_{r}}\right)$$

where *R_b_* is the reference slant range of block *b*. The range-frequency spectrum is then resampled according to the SSM relation given as $f_{\tau }^{\prime }=G_{b}\left(f_{c}+f_{\tau },f_{a}\right)-G_{b}\left(f_{c},f_{a}\right)$. After the range inverse Fourier transform, the remaining range-dependent azimuth phase is compensated by

(9) $$H_{\mathrm{AC},b}\left(f_{a};R\right)=\exp\left\{j\frac{4\pi \left(R-R_{b}\right)}{c}\left[G_{b}\left(F,f_{a}\right)-f_{c}\right]\right\}$$

where R denotes the slant range corresponding to the range bin. Finally, the azimuth inverse Fourier transform is performed to obtain the focused SAR image.

### 2.3. Image-Quality Prediction Using a One-Dimensional Azimuth-Focusing Kernel

For each evaluation cell, an azimuth impulse response function (IRF) is generated by the proposed one-dimensional azimuth-focusing kernel based on the reference function and the ideal SSM relation defined in Section 2.2. Azimuth resolution, PSLR, and ISLR are then evaluated from the resulting IRF and used as the principal image-quality parameters for initial block design.

The validity of the center-frequency approximation (*f_τ_* = 0) is examined by analyzing the range-frequency dependence of the residual phase. Consider point target p processed using the reference slant range *R_b_* and squint angle *θ_b_* of block b. Assuming ideal and distortion-free SSM, with *H_AC_* evaluated at the target range bin *R* = *R_p_* the residual phase at the output of the block-reference processing chain is given by

$$\;\Phi_{p|b}\left({F,f}_{a}\right)=-\frac{4\pi R_{p}}{c}\left[G_{p}\left(F,f_{a}\right)-f_{c}\right]\;\;\;\;\;+\frac{4\pi }{c}\left[R_{b}+(R_{p}-R_{b})\right]\left[G_{b}(F,f_{a})-f_{c}\right]\;\;\;\;\;=\frac{4\pi R_{p}}{c}\left[G_{b}(F,f_{a})-G_{p}(F,f_{a})\right]$$

(10)

Equation (10) includes both the range and squint-angle offsets between point target and the block reference. To examine the range-frequency dependence neglected by the center-frequency approximation, the residual phase in (10) is expanded with respect to *f_a_* as

(11) $$\Phi_{p|b}\left(F,f_{a}\right)=C_{0}\left(F\right)+C_{1}\left(F\right)f_{a}+C_{2}\left(F\right)f_{a}^{2}+O\left(f_{a}^{3}\right)$$

where

(12) $$C_{n}\left(F\right)={\left.\frac{1}{n!}\frac{\partial^{n}\Phi_{p|b}\left(F,f_{a}\right)}{\partial f_{a}^{n}}\right|}_{f_{a}=0}$$

The constant and linear terms introduce only phase and position shifts, whereas the quadratic term causes azimuth defocusing. Because the direct range-offset dependence is accommodated by the range-dependent azimuth-compression kernel, the following analysis focuses on the residual defocusing caused by the squint-angle mismatch, while retaining the target slant range R = R_p_. The quadratic coefficient is then given by

(13) $$C_{2}\left(F,\Delta \theta \right)=\frac{\mathrm{\pi Rc}\left[\cos\left(\theta_{b}+\Delta \theta \right)-\cos\theta_{b}\right]}{2V^{2}\left(f_{c}+f_{\tau }\right)\cos^{3}\theta_{0}}$$

The 1D method is evaluated at the center range frequency, *f_τ_* = 0, giving

(14) $$C_{2}^{1D}\left(f_{c},\Delta \theta \right)=\frac{\mathrm{\pi Rc}\left[\cos\left(\theta_{b}+\Delta \theta \right)-\cos\theta_{b}\right]}{2V^{2}f_{c}\cos^{3}\theta_{0}}$$

The range-frequency-dependent coefficient is therefore related to its center-frequency value by

(15) $$C_{2}\left(F,\Delta \theta \right)=C_{2}^{1D}\left(f_{c},\Delta \theta \right)\frac{f_{c}}{f_{c}+f_{\tau }}$$

For a processed range bandwidth *B_r_*, the relative variation in the quadratic coefficient is

(16) $$\left|\frac{C_{2}\left(F,\Delta \theta \right)}{C_{2}^{1D}\left(f_{c},\;\Delta \theta \right)}-1\right|=\left|\frac{f_{\tau }}{f_{c}+f_{\tau }}\right|\;$$

Over −*B_r_*/2 ≤ *f_τ_* ≤ *B_r_*/2, the maximum occurs at *f_τ_* = −*B_r_*/2, because *f_c_* + *f_τ_* reaches its minimum value. Therefore,

(17) $$\epsilon_{C_{2},\max}=\frac{B_{r}/2}{f_{c}-B_{r}/2}\approx \frac{B_{r}}{2f_{c}},B_{r}\ll f_{c}$$

For the simulation parameters in this study, where the processed range bandwidth is 150 MHz and the wavelength is 0.03 m, corresponding to a carrier frequency of approximately 10 GHz, yielding a maximum relative variation of approximately 0.76% in the residual quadratic-phase coefficient. This limited variation indicates that the center-frequency 1D azimuth response can closely represent the azimuth resolution, PSLR, and ISLR obtained from the full ω–k chain under the modeled conditions. The residual-phase analysis therefore provides the theoretical basis for applying the 1D approximation to the initial block-design process.

### 2.4. Estimation of the Evaluation-Grid Resolution

From (14), the dominant residual azimuth phase caused by the squint-angle mismatch is used to determine an appropriate subdivision of the imaging region for subsequent performance prediction.

(18) $$\phi_{2,\max}\left(f_{c},\Delta \theta \right)=\left|C_{2}^{1D}\left(f_{c},\Delta \theta \right)\right|{\left(\frac{B_{a}}{2}\right)}^{2}$$

For an allowable phase error *ϕ*_2,th_, the angular extent is obtained from

(19) $$\left|\cos\left(\theta_{b}+\Delta \theta \right)-\cos\theta_{b}\right|\leq \Gamma_{b},\Gamma_{b}=\frac{8V^{2}\cos^{3}\theta_{0}}{\pi R_{b}\lambda B_{a}^{2}}\phi_{2,\mathrm{th}}$$

The corresponding full ground extent, accounting for both negative and positive angular offsets, is approximated by

*W*_g_ = 2*R*_b_cos*θ*_b_|Δ*θ*_max_| (20)

Table 1 gives the maximum allowable squint-angle mismatch and the corresponding ground extent for representative phase-error limits using the simulation parameters in this study.

### 2.5. Oblique Phase-Error Contours

Because the quadratic residual phase depends jointly on range and squint angle, its constant-error boundaries satisfy

(*R_b_*+Δ*R_b_*_,*p*_)[cos(*θ_b_*+Δ*θ_b_*_,*p*_) − cos*θ_b_*] = ±*R_b_Γ_b_* (21)

where Δ*R_b_*_,*p*_ = *R_p_* − *R_b_* and ∆*θ_b_*_,*p*_ = *θ_p_* − *θ_b_*. For a small angular offset, (21) reduces to

(22) $$\Delta \theta_{b,p}\approx \mp \frac{R_{b}\Gamma_{b}}{\sin\theta_{b}\left(R_{b}+\Delta R_{b,p}\right)}\;$$

For local analysis, consider a point (ΔR*_c_*, Δθ*_c_*) on either constant-error boundary. A first-order Taylor expansion of the boundary about this point gives

(23) $$\Delta \theta_{b,p}\approx \Delta \theta_{c}-\frac{\Delta \theta_{c}}{R_{b}+\Delta R_{c}}\left(\Delta R_{b,p}-\Delta R_{c}\right)\;$$

As shown in (23), the squint-angle offset varies linearly with the range offset with a nonzero slope. Therefore, the constant-error contours are oblique in the range–squint plane. This result explains why the spatial extent satisfying a specified image-quality requirement can also exhibit an oblique distribution.

### 2.6. Image-Quality Prediction for Block-Processing Planning

For initial block-design planning, a cell is considered to satisfy the specified image-quality condition when its azimuth-resolution degradation does not exceed 5% and its PSLR and ISLR degradations remain within 3 dB of the reference response. The thresholds were selected as representative planning tolerances by considering image-quality levels typically considered acceptable for practical SAR image interpretation. These values are not intended to represent universal block-validity criteria and can be selected by the user according to the required image quality and system requirements.

For each candidate processing reference, the region satisfying all three image-quality criteria is determined from the predicted azimuth resolution, PSLR, and ISLR. This region is regarded as the feasible block extent for that processing reference.

- Evaluation-grid partitioning: Divide the imaging region into evaluation cells with a spacing determined from the phase-error-based ground extent in Table 1.
- Candidate-reference selection: Select a candidate processing-reference cell.
- Quality-valid-region prediction: Predict the azimuth IRFs at the evaluation cells and identify the cells satisfying the resolution, PSLR, and ISLR criteria.
- Planning-map update: Record the predicted quality-valid region and evaluate additional candidate references as required.
- Termination: Continue the evaluation until the imaging region has been sufficiently characterized for initial block design.

Unlike a partitioning approach based only on a prescribed phase-error bound, the proposed approach evaluates the predicted azimuth impulse response at each cell and expresses the result directly in terms of resolution, PSLR, and ISLR. The resulting planning map can therefore be used to compare candidate processing references and estimate feasible block extents before block design with the real SAR data.

The evaluation-grid size should be selected according to the image width, acquired-image resolution, and squint angle. In general, a larger image width, a finer acquired-image resolution, and a larger squint angle can require a denser evaluation grid and a larger number of candidate processing references, thereby increasing the total computational load. The proposed method enables these design parameters to be rapidly explored during the initial block-design stage.

To evaluate the computational efficiency of this initial block-design procedure, the execution times of both the proposed method and full ω–k processing were measured in MATLAB R2023b on a PC equipped with a 2.1-GHz Intel Core i7-13700 processor and 32 GB of RAM. Table 2 reports the total measured execution time required to evaluate the specified candidate references for each evaluation-grid size.

For the 21 × 21 evaluation grid, the proposed method requires 41 s to evaluate 21 candidate processing references, whereas full ω–k processing requires 1827 s for the same set of references. The corresponding computation-time reduction factor is approximately 44.6. For the 31 × 31, 41 × 41, 51 × 51, and 61 × 61 evaluation grids, the measured reduction factors are approximately 22.9, 12.1, 8.6, and 5.5, respectively.

The full ω–k processing time for a single candidate reference consists of 12.546 s for preprocessing, including the 2D FFT, 72.440 s for RFM–SSM–range IFFT, and 1.918 s for azimuth compression and azimuth IFFT, giving a total of approximately 86.9 s. Although the relative speedup decreases as the evaluation grid becomes denser, the proposed method requires substantially less computation than repeated full ω–k processing for all grid sizes in Table 2. These timing results apply to the initial design stage under idealized simulation conditions and may differ from those of the final block design using real SAR data.

## 3. Distributed Point-Target Simulation and Validation

### 3.1. Simulation Setup

**Figure 3:**
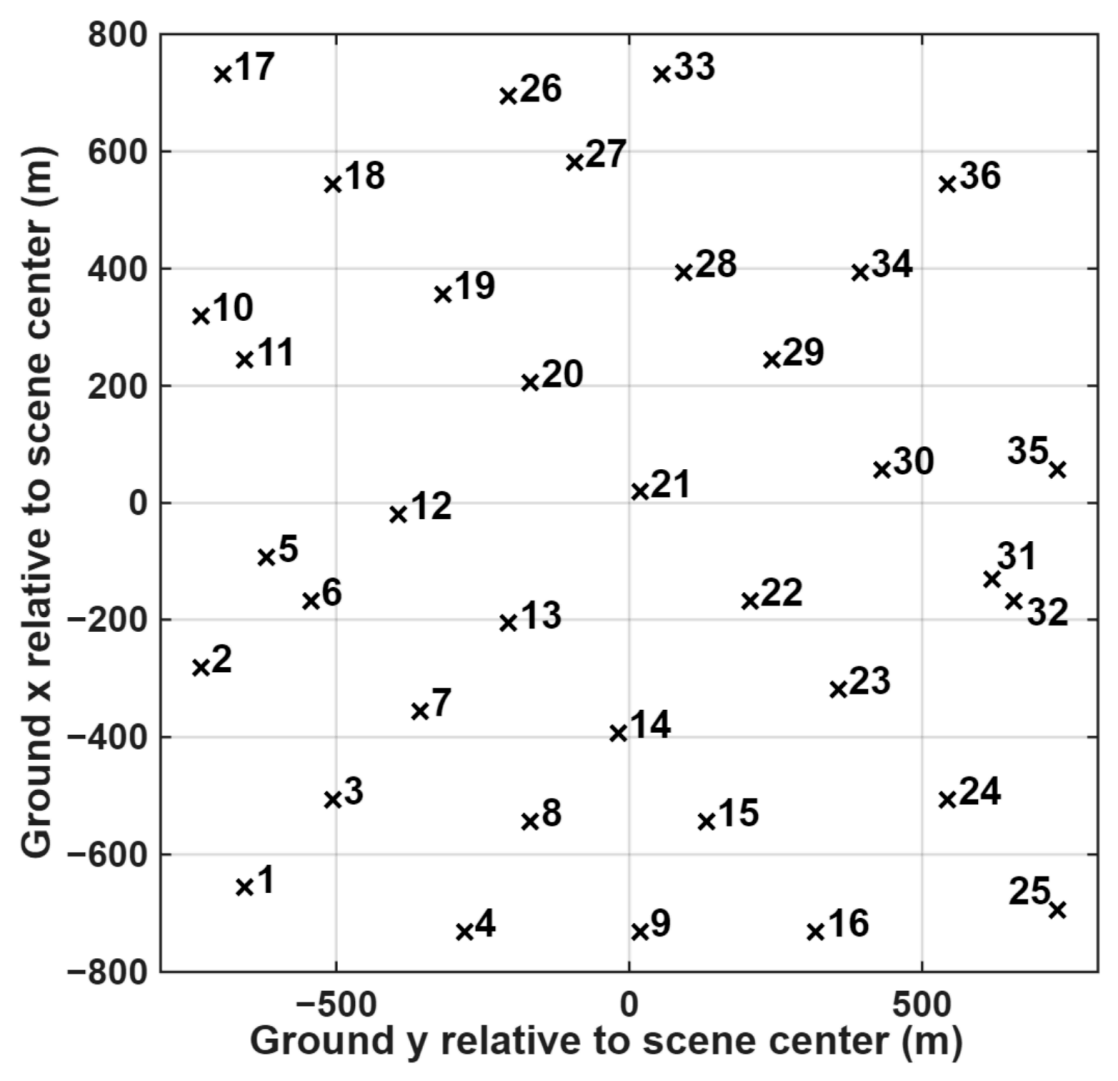
Spatial distribution and IDs of the 36 point targets over the 1.5 km × 1.5 km ground scene. Coordinates are relative to the scene center.

A 1.5 km × 1.5 km ground scene is divided into a 41 × 41 evaluation grid. Thirty-six point targets are distributed in multiple oblique diagonal groups to cover a broad range of slant-range and squint-angle offsets. Four candidate processing references, B1–B4, are selected along the opposite diagonal of the grid, and the image-quality-valid region is determined for each reference. Each point target is then associated with the processing reference to which it belongs, and the azimuth resolution, PSLR, and ISLR are compared with those obtained from the full ω–k processing. The same target geometry is used to generate the raw data for the full ω–k processing. The principal simulation parameters are summarized in Table 3. Figure 3 shows the spatial distribution and target IDs in ground coordinates relative to the scene center. The 36 targets span both positive and negative azimuth and ground-range offsets, enabling validation over a broad portion of the 1.5 km × 1.5 km test area.

### 3.2. Simulation Results

**Figure 4:**
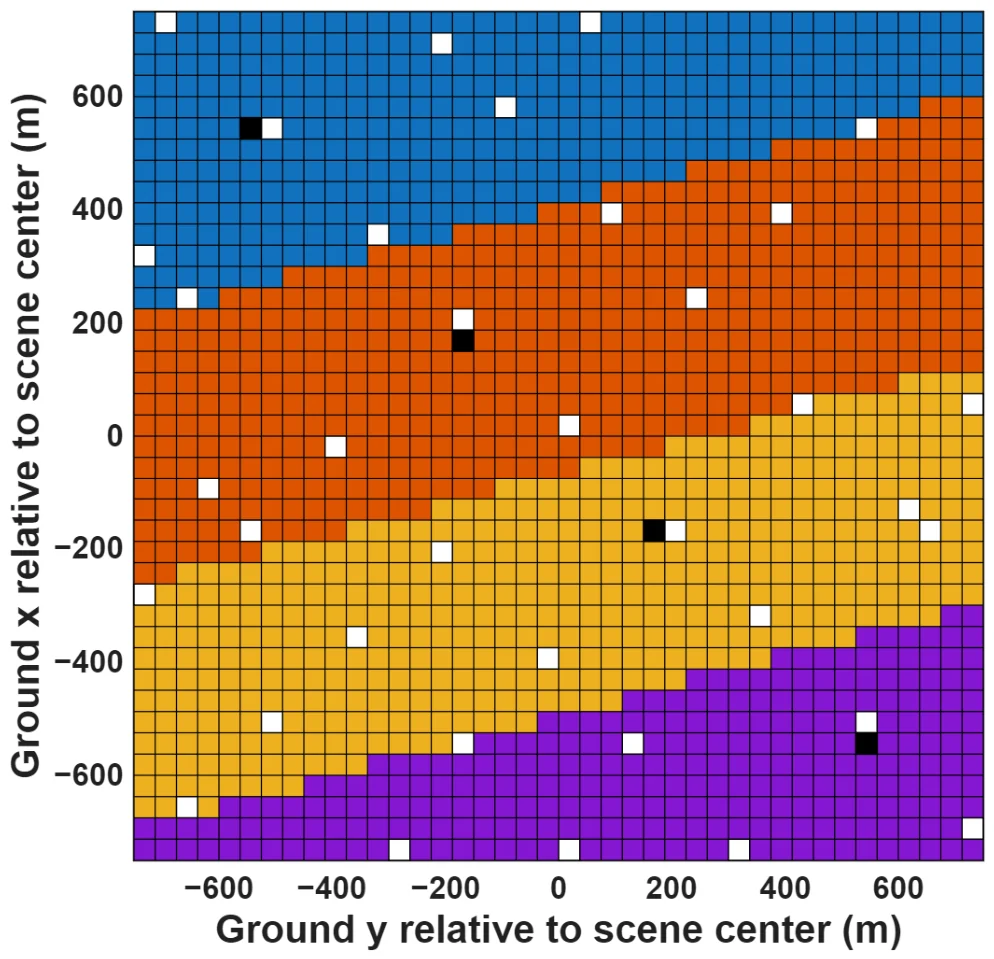
Image-quality-valid regions for the four processing references over the 41 × 41 evaluation grid. Colored regions indicate the areas satisfying the specified image-quality criteria, where each distinct color represents a region sharing the same level of image quality. The black cells denote the reference points.

Figure 4 shows the image-quality-valid regions obtained for the four candidate processing references over the 41 × 41 evaluation grid. For each processing reference, the corresponding boundary defines the region within which the specified azimuth-resolution, PSLR, and ISLR criteria are satisfied. Unlike our intuitive expectation that cells at similar distances from a processing reference would exhibit similar focusing performance, Figure 4 shows that the performance-valid boundaries form oblique patterns rather than symmetric regions around the reference locations. As theoretically analyzed in Section 2.5, the range offset and squint-angle mismatch are coupled with respect to each processing reference. This coupling produces oblique constant-error contours, and the simulation results show the same oblique characteristic in the image-quality-valid boundaries. The white cells indicate the 36 point targets used for validation, whereas the black cells indicate the four processing-reference locations.

**Figure 5:**
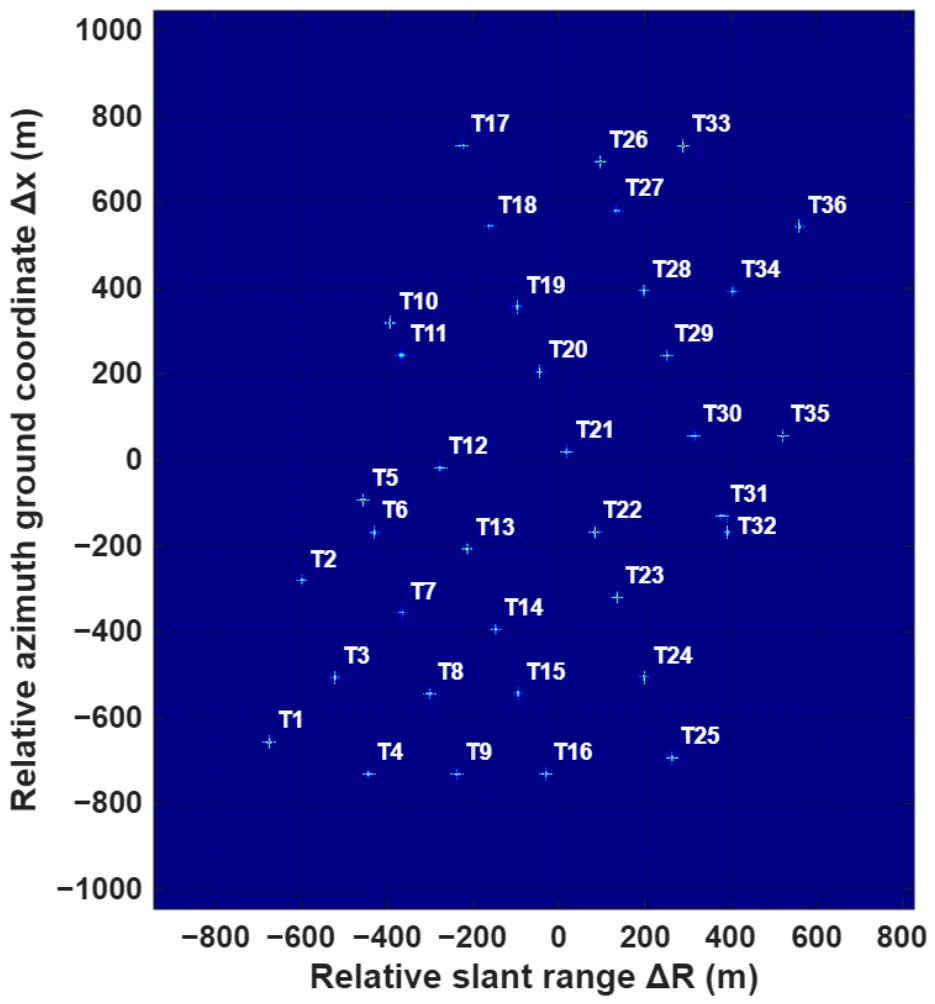
Focused image of the 36 point targets obtained using full ω–k processing.

To validate whether the image quality predicted using only the azimuth-focusing kernel is consistent with that obtained from conventional two-dimensional SAR processing, the same point-target data are also processed using the full ω–k algorithm. The azimuth resolution, PSLR, and ISLR measured from the full ω–k focused images are then compared with the corresponding predicted values. Figure 5 shows the resulting full ω–k focused image of the 36 point targets. The image is displayed in the processing-coordinate plane, where the horizontal axis represents the relative slant range, Δ*R*, and the vertical axis represents the relative azimuth ground coordinate, Δ*x*. The focused target locations correspond to the target distribution shown in Figure 3.

### 3.3. Comparison of Image Quality Between Proposed Method and Full ω–k Processing

**Figure 6:**
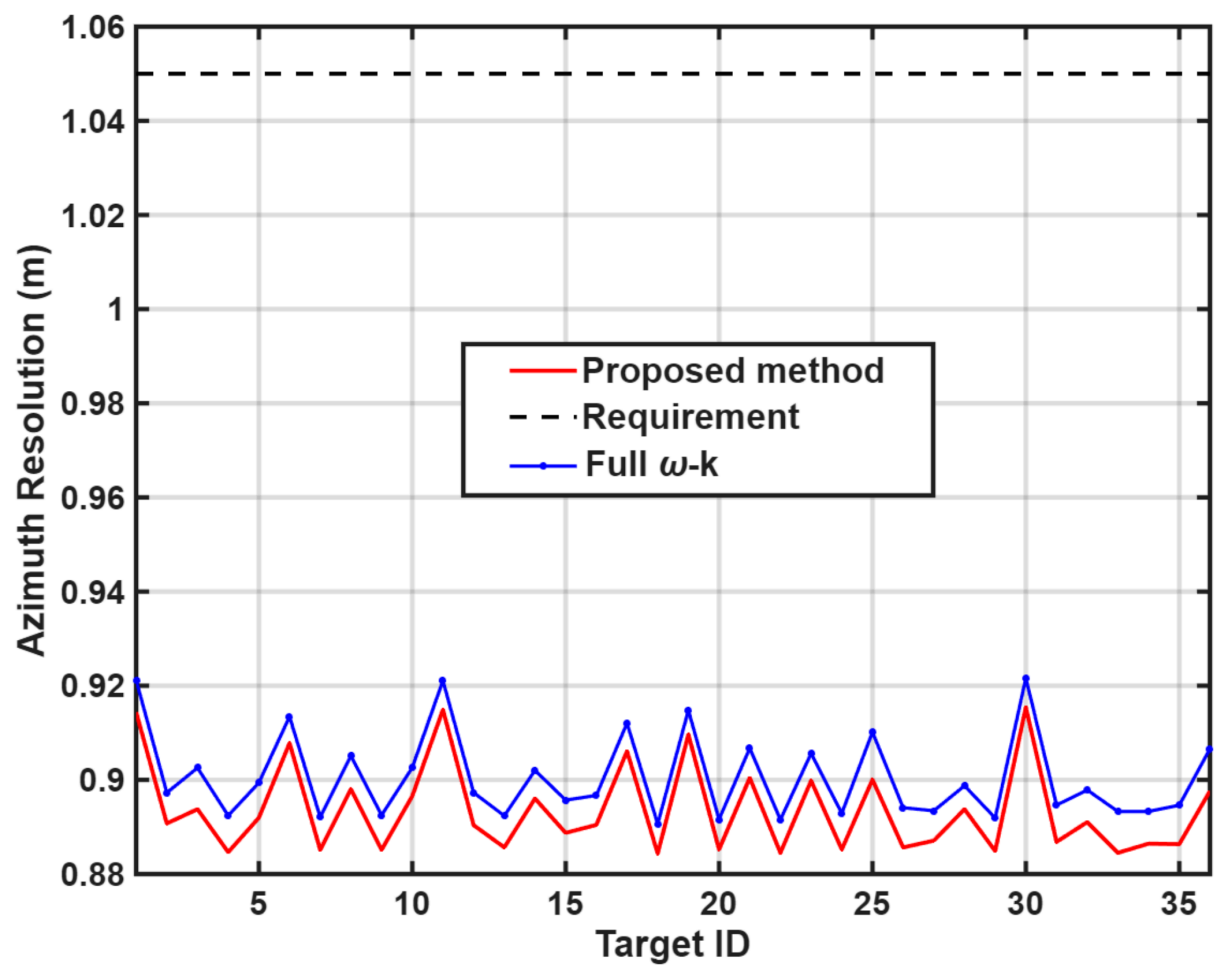
Comparison of azimuth resolution obtained using the proposed method and the full ω–k algorithm for the 36 point targets.

**Figure 7:**
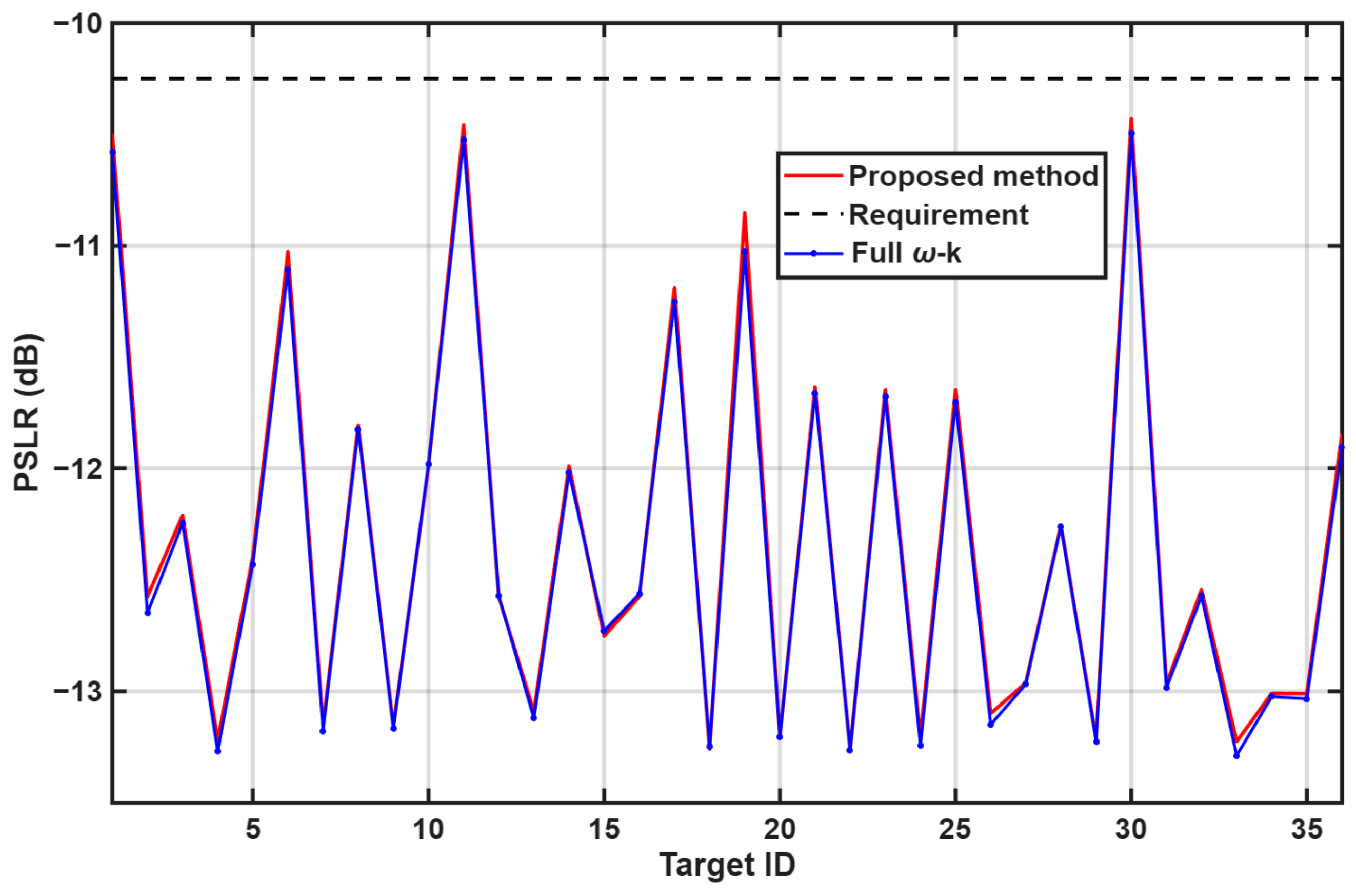
Comparison of PSLR obtained using the proposed method and the full ω–k algorithm for the 36 point targets.

**Figure 8:**
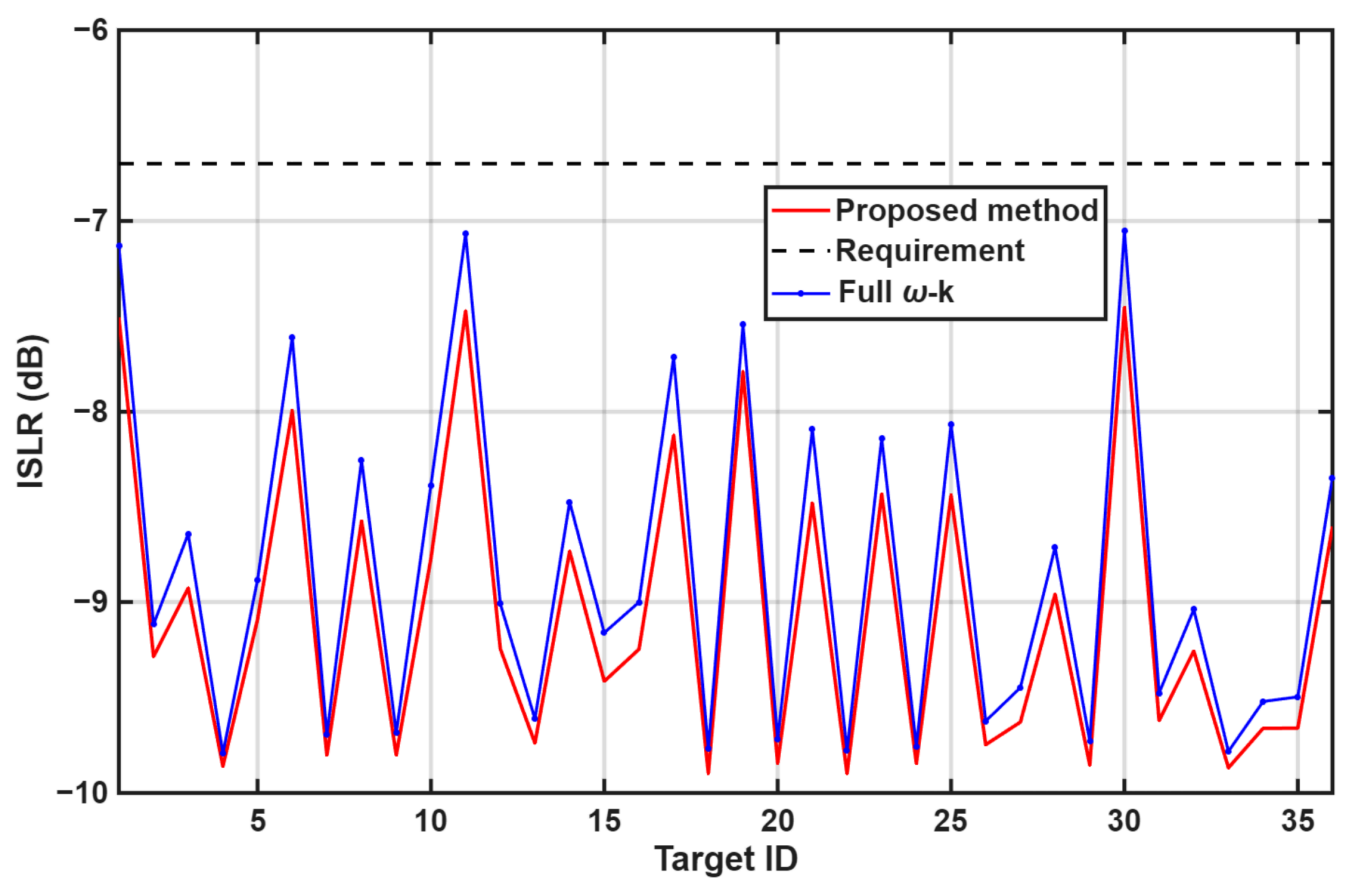
Comparison of ISLR obtained using the proposed method and the full ω–k algorithm for the 36 point targets.

Using the focused target responses in Figure 5, Figure 6, Figure 7 and Figure 8 compare the predicted azimuth resolution, PSLR, and ISLR with the corresponding values measured from the full ω–k processing for all 36 targets. The specified image-quality limits are shown together with both results to evaluate not only the agreement in metric values but also whether the same quality criteria are satisfied. The comparison evaluates the agreement between the image-quality results obtained using the proposed method and those obtained using the full ω–k algorithm.

Figure 6 shows that the azimuth-resolution results obtained using the proposed method closely agree with those obtained using the full ω–k algorithm. The root-mean-square error (RMSE) for azimuth resolution is 0.007046 m. Figure 7 and Figure 8 show similarly close agreement for PSLR and ISLR, with RMSE values of 0.049759 dB and 0.251709 dB, respectively. All 36 targets yield the same pass/fail decisions for the specified image-quality criteria using both methods.

The present validation is limited to idealized simulation conditions and does not include distributed scattering, topographic variation, motion and navigation errors, calibration errors, or other effects present in real SAR data. Therefore, the results demonstrate the accuracy of the proposed method for initial block-design analysis under the modeled geometry. For final block design using real SAR data, additional scene-dependent effects and practical margins may need to be considered.

In real SAR data, distributed scattering and clutter may make the impulse-response characteristics less directly observable than in isolated point-target simulations and may therefore introduce uncertainty in the evaluation of azimuth resolution, PSLR, and ISLR. Terrain variations can change the local slant range and imaging geometry from those assumed in the nominal model. In addition, motion and navigation errors, calibration errors, and other non-ideal system effects can introduce additional phase errors that are not represented in the present one-dimensional prediction. These effects may therefore cause the image quality obtained from real SAR data to differ from the nominal performance predicted during the initial design stage.

The initially predicted block-design parameters can be used as practical starting values when processing actual SAR data. Any changes in the block configuration caused by distributed scattering, clutter, terrain variation, motion and navigation errors, calibration errors, or other real-data effects must then be identified and accounted for during the final block partitioning based on the actual SAR data, see Table 4.

## 4. Conclusions

This study investigated whether the computationally expensive full ω–k processing kernel used for initial block-design analysis can be replaced by a simple one-dimensional azimuth-focusing kernel. The feasibility of this dimensional reduction was first examined theoretically and was then validated through point-target simulations. The results showed that the proposed approach can reproduce the image-quality trends obtained from full ω–k processing with sufficient accuracy for initial block-design planning.

An interesting observation from this study is that, for squinted SAR imaging, processing-reference locations are better arranged along an oblique or diagonal direction than on a uniformly partitioned grid. As theoretically analyzed in this study, the range offset and squint-angle mismatch are coupled with respect to each processing reference. This coupling makes the constant-error contours oblique. The simulation results confirm this behavior. Regions with similar focusing performance form tilted boundaries rather than symmetric regions around the processing reference.

Based on this result, the proposed method can provide practical guidance before final block design is performed using real SAR data. Virtual point targets can be distributed over the entire imaging area to predict their azimuth performance before the actual block design. These predicted results can then provide practical guidance for selecting the processing-reference locations, block extents, and other design parameters when block design is performed using real SAR data.

In the present validation, the independently processed point-target responses were placed at their corresponding ground positions in a common ground-coordinate image using a simplified mosaicking procedure. Because isolated point targets were used, detailed overlap weighting and block-to-block registration were not required. For complete block-based reconstruction using real SAR data, however, adjacent blocks with oblique valid boundaries will require appropriate overlap handling and transformation or resampling into a common output coordinate system before final mosaicking. These reconstruction procedures remain subjects for future work.

Because this initial design analysis can be performed without repeatedly executing the full ω–k algorithm, the proposed approach substantially reduces the computational burden of candidate-reference evaluation. The method therefore provides a practical and computationally efficient means of establishing an initial block-design guideline for squinted spotlight SAR imaging.

## Figures and Tables

**Table 1:** Maximum allowable squint-angle mismatch and corresponding ground extent.

*ϕ*_2,*th*_	|*Δ*θ|_max_ (Deg)	Ground Extent *W_g_* (m)
π/16	0.00292	76.0
π/12	0.00389	101.3
π/8	0.00584	152.0
π/6	0.00779	202.6
π/4	0.01168	303.9
π/3	0.01557	405.1

**Table 2:** Block-design execution time.

Evaluation Grid	Candidate References (Diagonal Cells)	1D Prediction Time (s)	Full ω–k Time (s)
21 × 21	21	41	1827
31 × 31	31	118	2697
41 × 41	41	296	3567
51 × 51	51	513	4437
61 × 61	61	960	5307

**Table 3:** Point-target simulation parameters.

Parameter	Value
Platform altitude/speed (*h*/*V*)	510 km/7.2 km/s
Wavelength (*λ*)	0.03 m
Squint angle (*θ*_0_)/Grazing angle (*γ*)	20°/40°
Reference slant range (*R_ref_*)	793.4 km
Azimuth processing bandwidth (*B_a_*)	7200 Hz
Range processing bandwidth (*B_r_*)	150 MHz
Nominal range/azimuth resolution	1 m
Scene size	1.5 km × 1.5 km
Evaluation grid	41 × 41 cells
Point targets	36
Processing reference blocks	4 (B1–B4)
Quality criteria	Resolution deg. ≤ 5%; PSLR/ISLR deg. ≤ 3 dB

**Table 4:** Summary of the agreement between the proposed method and the full ω–k algorithm for the 36 point targets.

Metric	Result
Azimuth-resolution RMSE	0.007046 m
PSLR RMSE	0.049759 dB
ISLR RMSE	0.251709 dB
Pass/fail agreement	36/36 targets (100%)

## Data Availability

The data presented in this study are available on request from the corresponding author.
